# Targeting Uterine Quiescence: A Multitarget Strategy with Vitamin D, High Molecular Weight Hyaluronic Acid, Magnesium, and Palmitoylethanolamide to Prevent Preterm Birth

**DOI:** 10.3390/nu18010113

**Published:** 2025-12-29

**Authors:** Ilenia Mappa, Giuseppina Porcaro, Martina Derme, Giuseppe Rizzo

**Affiliations:** 1 Department of Maternal and Child Health Urological Sciences, Sapienza University, 00161 Rome, Italy; 2 Department of Gynecology and Obstetrics, Women’s Health Centre, 05100 Terni, Italy

**Keywords:** high molecular weight hyaluronic acid, vitamin D, palmitoylethanolamide, physiological pregnancy, premature contractility prevention, PTB

## Abstract

Maintaining a quiescent uterus until labor is of utmost importance for a successful pregnancy and still represents the most challenging issue in clinical practice. Despite the existence of standard approaches (short-term use of tocolytic agents or preventive use of vaginal progesterone), whose efficacy is still controversial, several natural molecules have garnered attention in recent years as an effective therapeutic approach in high-risk pregnancies and beyond. Despite inflammatory activation, premature contractility depends on several factors, since myometrial quiescence is a complex mechanism not fully understood. Therefore, the synergistic activity of different natural molecules could be an innovative approach for acting simultaneously and maintaining uterine quiescence.

## 1. Introduction

Maintaining a quiescent uterus until labor onset is of utmost importance for the development and maturation of the fetus. The length of pregnancy is tightly regulated to ensure the delivery of a newborn mature enough to survive in the extra uterine environment. Unfortunately, when myometrium contractility is prematurely activated, there is asynchrony between the start of labor and fetal development, and neonatal complications may occur [1]. According to a recent cross-sectional study, 68% of analyzed participants experienced at least one pregnancy complication, including preterm birth (PTB) [2], which still represents the major health problems in obstetrics, with an estimated 13.4 million (95% credible interval [CrI] 12.3–15.2 million) newborn babies born preterm (<37 weeks) in 2020 (9·9% of all births [95% CrI 9.1–11.2]) [3] Consequently, myometrial quiescence maintenance and avoidance of premature myometrial activation still represents the most challenging issue that clinicians encounter in their clinical practice.

Currently, the first line approach in the case of premature uterine contractility is the use of tocolytic drugs (β-agonists, magnesium sulfate, calcium channel blockers, prostaglandin synthetase inhibitors, NO donors, and tractocile), whose use is still controversial [4]. Short-term use of tocolytics may delay delivery for at least 24–48 h, allowing for administration of antenatal steroids and other therapies to reduce neonatal mortality and multiple morbidities [5]. Unfortunately, there is a lack of evidence to suggest that tocolysis may maintain pregnancy for a longer period; thus, most of these agents are often questioned [6,7]. Many of them, for example, are unsuitable during preterm labor due to vaginal bleeding, ruptured membranes, multiple gestation, or advanced cervical dilatation [8]. The number of tocolytics in current usage highlights that no ideal tocolytic exists, with some having significant long-term cardiovascular system, metabolic, and neuromuscular adverse effects. The selection of tocolytic therapy remains debatable due to differences in availability, cost, safety, and efficacy [4,9].

Progestogens have been extensively researched as a means of preventing PTB in different clinical conditions, but their real efficacy is still up for debate. Women with singleton pregnancies with mid-trimester short cervixes who received vaginal progesterone had a considerably lower incidence of PTB before 33 weeks of gestation, but there were no appreciable differences in fetal or perinatal mortality [10]. On the contrary, vaginal progesterone in the optimum randomized trial did not affect PTB nor neonatal outcome [11]. Another randomized controlled trial concluded that the use of progestogens in women with a short cervix did not reduce the rate of PTB [12]. These conflicting results demonstrate that a consensus on this argument has not yet been reached; therefore, further clinical trials are needed [13].

Birth can be considered an inflammatory event [14], with an upregulation of a large panel of pro-inflammatory genes, both in fetal membranes and myometrium, occurring with labor onset [15]. Pro-inflammatory signals build up and are amplified by positive feedback interactions involving paracrine and autocrine systems at the level of each intrauterine cell and tissue, causing the change from a quiescent to an active state [14].

Nuclear factor-kappa B (NF-κB) is a redox-sensitive transcription factor, playing a pivotal role in the expression of a variety of genes involved in inflammatory responses and apoptosis in multiple tissues and cell types [16]. It is one of the master regulators in the shift from a quiescent status to an active/contractile status. For instance, amnion, “activated” for labor, is characterized by a high NF-κB activity and exhibits an increase in the expression of several pro-inflammatory and pro-contractile genes [17]. The premature or aberrant action of NF-κB may contribute to uterine contractility, thus leading to PTB [18]. NF-κB regulates a complex network of signaling pathways involved in amnion activation, such as interleukin 1β (IL-1β) and interleukin 6 (IL-6), which are involved in switching feto/maternal tissue activity for labor.

At term labor, IL-1 β is upregulated in amniotic fluid and intrauterine tissues, including myometrium, decidua, cervix and fetal membranes [19,20].

Moreover, through the activation of cyclooxygenase-2 (COX-2), IL-1β is a powerful activator of prostaglandin production in all gestational organs [21]. A study conducted on the European population reported that IL-1β was positively and significantly associated with preterm delivery. Specifically, for every unit increase in IL-1β, women were, on average, 7.2 (OR: 7.2, CI: 1.94–26.77, *p* = 0.003) times more likely to deliver preterm, thus making IL-1β a predictive marker of preterm labor (PTL) [22].

One of the key mediators stimulated by IL-1β is IL-6, a pro-inflammatory cytokine widely expressed in the decidual tissue, placenta, fetal membrane and amniotic fluid [23], and involved in a wide range of biological functions, including implantation, pregnancy, and parturition [14]. Its production is increased by myometrium, cervix, and choriodecidua before and during labor and elevated or deficient IL-6 levels are associated with infertility, recurrent miscarriage, pre-eclampsia and preterm delivery [24]. Even in the second trimester, women who gave birth spontaneously before 35 weeks had noticeably higher levels of IL-6 in their amniotic and cervical fluid [25,26]. Therefore, the inhibition of these pro-inflammatory factors has raised attention as a possible clinical strategy to inhibit premature uterine contractility.

The aim of this review is to focus on different natural molecules and their potential synergic activity to maintain uterine quiescence.

## 2. Methods

Although no formal systematic search was conducted, the referenced studies were primarily identified through major databases, including PubMed, Scopus, and Web of Science, carried out in accordance with the Preferred Reporting Items for Systematic Reviews and Meta-Analyses (PRISMA) criteria. Research that examined the impact of vitamin D, magnesium, high molecular weight hyaluronic acid (HMWHA), palmitoylethanolamide (PEA) and other natural molecules on pathways associated with uterine quiescence, inflammation in gestational tissues, or the prevention of preterm birth (PTB), was suitable for inclusion criteria. Three categories of studies were covered in the review:

**In vitro research**: molecular pathways (such as NF-κB, cytokine expression, and contractile protein expression) are reported using cell culture models (such as myometrial smooth muscle cells, amniotic cells, and immune cells).
**Animal studies**: in vivo models (such as rat models of inflammation-induced preterm birth) assessing the target molecules’ effectiveness in lowering inflammatory indicators or preventing premature delivery.
**Clinical studies**: human studies that report on pregnancy outcomes (e.g., preterm birth, uterine contractions, cervical length, subchorionic hematoma resorption) or pertinent biomarkers (e.g., plasma cytokine levels) include randomized controlled trials (RCTs), prospective/retrospective cohort studies, and case-control studies.

The search strategy used a combination of controlled vocabulary (e.g., MeSH terms) and free-text keywords related to the four target molecules and relevant outcomes. Given the narrative scope, study quality was assessed informally based on experimental design, without applying standardized risk-of-bias tools.

## 3. Natural Molecules for Preventing Premature Contractility

### 3.1. Vitamin D

Due to its immunomodulatory action, by promoting immune tolerance, vitamin D has a peculiar role in supporting pregnancy at different gestational stages [27,28,29,30]. Certainly, its most important role is that of being a “progesterone-like hormone”, exerting similar activities to progesterone (P4), in different phases of the menstrual cycle [31,32]. P4 has a role in the various stages of pregnancy, and its immunomodulatory effects are mediated through the protein progesterone-induced blocking factor (PIBF). PIBF is a 34 kDa immunosuppressive factor that induces a Th2-dominant cytokine response and modulates natural killer (NK) cell activity in a cytokine-mediated way [33,34]. PIBF urinary levels are significantly lower in women with threatened miscarriage, PTB, or in patients with preeclampsia, compared to women with physiological pregnancy [35], thus indicating that it is required for the maintenance of physiological pregnancy [36]. PIBF represents the link between P4 and vitamin D immunomodulatory action. Phytohemagglutinin (PHA)-activated peripheral lymphocytes from healthy women in the follicular and luteal phases of the cycle were exposed to escalating levels of vitamin D and a standard dose of progesterone. The findings showed that peripheral cells treated with increasing vitamin D concentrations and a standard P4 concentration produced noticeably more PIBF than those treated with comparable vitamin D concentrations without progesterone [37].

Vitamin D shifts the inflammatory profile to a Th2/Treg profile, decreasing levels of interferon (IFN)-γ, IL-6, IL-17, IL-22, IL-23 and tumor necrosis factor (TNF)-α, increasing levels of anti-inflammatory cytokines IL-10 and TGF-β [38]. Vitamin D regulates decidual immunity by stimulating Treg cells through the expression of the forkhead box 3 (FOXP3) protein, which supports differentiation and expansion [39,40]. Moreover, it plays an important role also in preventing PTB, since its treatment decreases inflammation-induced cytokines and contractile-associated factors in the uterine myometrial smooth muscle cells through the NFκB pathway [41]. Vitamin D also has an important role in the control of uterine contractility. Calcitriol, in fact, inhibits the expression of the corticotropin-releasing hormone (CRH) and COX-2 genes in the placenta, whose levels are usually increased in women with PTB. CRH stimulates the production of metalloproteinase (matrix metalloproteinase 9-MMP9), which degrades fetal membranes, causing their rupture, and COX-2 synthesizes prostaglandins that induce cervical ripening and uterine contractions [42].

In the myometrium, vitamin D decreases the production of contractile-associated proteins and inflammatory indicators, including prostaglandin receptors and connexin 43, in a dose-dependent manner [43,44].

In 2015, Singh and colleagues demonstrated the role of vitamin D in reducing the risk of PTB. The study included 100 healthy women during their first pregnancy, with a gestational age between 12 and 16 weeks. Women were then divided into two groups: 50 women took 2000 U.I. per day of vitamin D until the end of pregnancy, while the other 50 women received no treatment. The mean gestational age in pregnant women with 2000 IU vitamin D supplementation per day was significantly improved (*p* < 0.05) compared to the control group. Moreover, PTB in cases was significantly low (8%) (*p* < 0.001) [45]. The impact of hypovitaminosis on PTB has also been evaluated in a recent meta-analysis [46]. Results evidenced that maternal circulating 25-OHD deficiency (pooled OR, 1.25; 95%CI: 1.13–1.38) rather than insufficiency (pooled OR, 1.09; 95%CI: 0.89–1.35) was associated with an increased risk of PTB, and vitamin D supplementation alone during pregnancy could reduce the risk of PTB (pooled RR, 0.57; 95%CI: 0.36–0.91) [47]. These findings were confirmed by another meta-analysis on 10.098 participants, thus evidencing that pregnant women with vitamin D deficiency (maternal serum 25 (OH) D levels < 20 ng/mL) experienced a significant risk of PTB (OR = 1.29; IC 95%: 1.16–1.45) [48]. A recent systematic review and meta-analysis integrated the latest studies up to 2024, offering a robust, evidence-based assessment of vitamin D supplementation impact on maternal outcomes. By analyzing a total of 33 RCTs involving 10,613 participants, results confirmed that vitamin D supplementation alone significantly reduced preterm labor of 30% (RR = 0.70, 95% CI [0.51, 0.96], *p* = 0.0286) than controls [49]. This action of vitamin D is further supported by some clinical studies in which vitamin D has been associated with other natural molecules [50,51] (Table 1).

### 3.2. High Molecular Weight Hyaluronic Acid (HMWHA)

High molecular weight hyaluronic acid (HMWHA) has emerged in recent years as a pivotal molecule to sustain a physiological pregnancy, from conception to full gestation, since it is either a structural or a regulatory molecule [52]. HMWHA can support the activity of endogenous progesterone (P4) [53], a key regulator of membrane homeostasis during physiological gestation. P4 inhibits myometrial contractility through genomic and non-genomic pathways [54]. Progesterone receptor membrane component 1 (PGRMC1) is engaged in cellular homeostasis and steroidogenesis, among other biological processes [55]. Several pieces of evidence also suggest that PGRMC1 mediates P4’s inhibitory effect on the contractility of the myometrium [56]. It is expressed in the myometrium during pregnancy as well as in all the layers of fetal membranes, amnion, chorion, and decidua layers of the fetal membrane. PGRMC1 is essential in supporting pregnancy since its lower expression is associated with PTL and the preterm premature rupture of membranes (PPROM) with or without histological chorioamnionitis (HCA) [56,57].

HMWHA increases the expression of PGRMC1 in a time and concentration-dependent manner [53]. Zhao et al. proved that PGRMC1 expression and HMWHA levels in patients with primary ovarian insufficiency (POI) were correlated, and that PGRMC1 mRNA and protein levels were also substantially increased in cells treated with HA [53].

HMWHA might also reduce the risk of PTB by blocking bacterial ascent via the cervical canal. According to scientific research, the cervical epithelium’s structural disarray is linked to HMWHA depletion. This may allow bacteria to more easily ascend through the cervical canal, causing inflammation and/or infection and ultimately raising the risk of PTB [58,59,60]. HMWHA polymers shield toll-like receptors (TLRs), thereby preventing the activation of inflammatory cascades [61]. HMWHA could also work synergistically with PEA in inhibiting mast cells, since they express different Toll-like receptors on their surface [62].

According to Lee et al., a high concentration of HMWHA significantly reduced the production of nitric oxide (NO) by lipopolysaccharide (LPS)-stimulated macrophages, upregulated the expression of genes linked to anti-inflammatory responses (M2 phenotype), including TGF-β1, IL10, IL-11, and Arg1, and decreased the expression of genes associated with classically activated (M1) macrophages, including TNF-α, IL-6, and IL-1β [61].

In vivo studies evidenced that HMWHA treatment had a protective effect against inflammation-mediated PTB by efficiently downregulating the pro-inflammatory cytokines TNF-α and IL-1β [63].

Clinical evidence about the vital role that oral HMWHA plays in supporting pregnancy evolution has emerged in recent years. Data from over 250 pregnant women, aged between 25 and 41 years old, at their 7th week of pregnancy, were gathered for a retrospective observational study. Oral supplement tablets containing 200 mg HMWHA were administered to 200 pregnant women in total, along with other natural compounds such as alpha lipoic acid, magnesium, vitamin B6, and vitamin D. In comparison to the control group, the therapy group experienced a considerably decreased percentage of adverse events, including uterine contractions (*p* = 0.0394) and preterm birth (*p* = 0.0092), in addition to premature hospitalization (*p* < 0.0001), pelvic pain (*p* < 0.0001) and miscarriage [50]. HMWHA, besides being a valid support to prevent PTB, is well known to counteract adverse events such as miscarriage. A recent clinical study examined whether oral HMWHA could encourage subchorionic hematoma resorption (SCH) and alleviate associated symptoms in pregnant women who were at risk of miscarriage, including vaginal bleeding, abdominal pain, and uterine contractions. A total of 56 expectant mothers with gestational ages ranging from 6 to 13 weeks were included in the study. Participants were divided into two groups: a treatment group (n = 31) that received the same progesterone regimen plus HMWHA (200 mg) together with other natural chemicals for two weeks, and a control group (n = 25) that received vaginal progesterone (200 mg twice daily). Subchorionic hematoma area decrease, or complete elimination, was the study’s main result. Reductions in maternal subjective symptoms, including uterine contractions, vaginal bleeding, and pelvic pain, were also achieved. When HMWHA was combined with natural molecules, the resorption of subchorionic hematoma and the remission of symptoms were substantially faster than in the control group [51] (Table 2).

### 3.3. Magnesium (Mg)

Magnesium is an oligo-element playing a crucial role in more than 600 enzymatic reactions in the body [64]. Even though it is present in many foods, modern processing methods have effectively eliminated magnesium from most diets, leaving many individuals lacking in this mineral. Interestingly, some preclinical and clinical research has demonstrated that a magnesium deficit is linked to several different health issues, including complications during pregnancy [65,66]. The role of magnesium in pregnancy has been a matter of investigation for a long time. Several studies demonstrated benefit in magnesium supplementation, such as reduced frequency of spontaneous abortion, premature deliveries and pregnancy complications [67]. Magnesium deficiency has been associated with increased risk of preterm birth [68,69]. Magnesium sulfate suppresses contractile response in pregnant human myometrial strips at a pharmacologic concentration of 5 mmol/L in a fashion consistent with both extra and intracellular processes by reducing both intracellular calcium and calcium influx [70]. Magnesium downregulates inflammatory cytokine production, such as TNF-α and IL-6. Moreover, it reduces NF-κB activation and nuclear localization [71]. Moreover, magnesium deficiency is associated with hyperactivity of the muscle cells in the uterus, which may consequently increase the risk of spontaneous abortion, preeclampsia, and preterm birth [68,72]. Higher magnesium levels or magnesium supplementation during pregnancy are consistently associated with a decreased incidence of preterm delivery, according to accumulated data from ecological, observational, and interventional studies [68] (Table 3).

### 3.4. Palmitoylethanolamide (PEA)

Despite the lack of clinical research, several scientific findings indicate that Palmitoylethanolamide (PEA) may be a natural molecule with potential applications in this field. PEA is a bioactive endogenous lipid mediator resembling endocannabinoids (eCB), belonging to the N-acyl-ethanolamine (NAE) fatty acid amide family. It is synthesized locally as needed within the lipid bilayer and can be detected in all tissues, including the brain [73]. The peroxisome proliferator-activated receptor (PPAR-α) is the primary target of PEA action, but it can also act on novel cannabinoid receptors, G protein-coupled receptors 55 (GPR55) and 119 (GPR119), or indirectly activate cannabinoid receptors 1 and 2 (CB1 and CB2) by preventing the breakdown of the endocannabinoid, anandamide (AEA), a process known as the “entourage effect”. PEA additionally exerts a strong anti-nociceptive impact via activating and desensitizing the transient receptor potential vanilloid receptor 1 (TRPV1) channels. It accomplishes this through several methods, including the entourage effect and PPAR-a activation and possibly functioning as a regulator of allosteric [73]. It is a pleiotropic molecule with anti-inflammatory, analgesic, anticonvulsant, antimicrobial, antipyretic, antiepileptic, immunomodulatory and neuroprotective activities [74,75,76]. For these reasons, PEA is already clinically helpful in a wide range of different disorders, such as depression, Parkinson’s disease, autism, or stroke [77,78,79]. All relevant data from randomized controlled trials (RCTs) assessing the effectiveness and tolerability of PEA supplementation across human ailments in patient populations have recently been gathered and thoroughly addressed by a systematic review and meta-analyses. RCTs examining the effects of PEA on pain management and overall wellbeing metrics have produced the strongest data, and they also show high tolerance when compared to controls. In addition to alleviating symptoms, PEA has been shown to modify biomarkers that are either early in the course of an illness or contribute to its progression, indicating the possibility of disease modification. PEA is also used in gynecological fields to suppress visceral disturbances across human conditions, such as endometriosis, chronic pelvic pain syndrome, and primary dysmenorrhea [80]. One intriguing finding from all these assessments was that PEA offers favorable safety for both short-term and long-term treatment, with a biological profile that closely resembles human physiology and possible disease-modifying effects. Recently, the hypothesis that PEA could also be used in the obstetric field has emerged. In a recent clinical trial, a total of 221 pregnant women, at ‘high-risk’ for PTB (previous PTB, a family history of PTB, history of 2 or more LLETZ procedures, cervical length < 25 mm on transvaginal ultrasound scan) were recruited between 24 and 34 gestational weeks [81]. The authors first measured AEA plasma levels, which are usually known to increase by the third trimester, and found that, also in the case of threatened PTB, their levels increased. Additionally, on a total of 51 patients, the authors also measured PEA plasma levels, using ultra-high performance liquid chromatography-tandem mass spectrometry. They evidenced that its concentrations were statistically higher (*p* = 0.042) in the women who delivered preterm (15.25 ± 5.38 nM) than in the women who delivered at term (12.01 ± 6.32 nM). The goal of the multivariate analysis was to consider both plasma AEA and PEA concentrations, a predictive marker for increased risk of PTB [81]. It could be explained by the fact that the increase in PEA levels is evidenced in many other inflammatory models and represents an anti-inflammatory response that the body initiates to establish homeostasis. Also in this case, with PTB being an “inflammation syndrome”, high levels of PEA could be either an anti-inflammatory response of the body to delay labor or maintain high levels of AEA itself, due to the entourage effect [81]. Unfortunately, endogenous PEA may be generally insufficient to counter chronic allostatic load as seen in chronic inflammatory disorders. Therefore, as suggested by the authors, making exogenous administration could be a viable therapeutic strategy to top-up endogenous levels and restore altered homeostasis [73].

Scientific studies highlighted how PEA acts on various factors that control premature uterine contractility. First, it significantly reduces, alone or in combination with other natural molecules, NF-kB levels, and increases those of its inhibitor, IkB [82,83]. PEA can downstream several inflammatory response-related genes, including cytokines (TNF-α, Il-1β, and IL-6) and inflammasome-dependent inflammatory pathways (NLRP3), whose activity is linked to preterm labor [84].

Moreover, PEA significantly downregulates signal mediators, such as COX2 [85], and downstream prostaglandin E2, whose increase is well known to lead to labor [86]. Moreover, the fact that PEA is an endogenous component of amniotic fluid and fetal membranes adds interest to the discussions about PEA, thus creating new scenarios and research opportunities in the field of obstetrics [87,88].

The further peculiar role of PEA in the context of preventing PTB could also depend on its action on the mast cell (MC). Mast cells are effectors of the immune system and are derived from bone marrow stem cells [89]. They are often associated with pathological disorders involving IgE (such as asthma), and they play a role in tissue remodeling, blood vessel formation, the resolution of viral and bacterial infections, and acute and chronic inflammatory diseases. Distributed throughout the body, mast cells are also normally present in the uterus, at the myometrial level, in close association with collagen fibers, fibroblasts, and smooth muscle cells [90]. Pregnancy problems such as asthma, psoriasis, mastocytosis, and urticaria can result from uncontrolled increases in MC quantity and/or activation, even though physiological amounts of MCs have been demonstrated to positively influence pregnancy outcomes, at least in mouse models [91]. Mast cells are under hormonal control throughout the gestational period. Endogenous progesterone (P4) keeps them in a quiescent state by inhibiting histamine release [92]. On the contrary, near childbirth, estrogens, oxytocin, and CRH trigger its degranulation. The released mediators thus activate uterine contractility and cervical ripening, promoting labor. Their premature activation and degranulation cause activation of several enzyme pathways, including arachidonic acid conversion by COX and lipoxygenase into prostaglandins, thus inducing an increased premature contractility in uterine tissues from pregnant humans [93]. The direct link between mast cells and uterine contractility has also been demonstrated in a study on human myometrial strips. The study highlighted that mast cell degranulation activates uterine contractility. Myometrial strips from women who delivered at term (>37 weeks) and women who delivered preterm (<37 weeks) were incubated with compound 48/80, which can activate mast cells. In both cases, treatment with this substance increased the contractility of the myometrial strips, while treatment with sodium cromoglicate (a mast cell stabilizer, an inhibitor of degranulation) reversed this increase in contractility. The use of a histamine receptor antagonist and a COX inhibitor also inhibited the induced contractility, both in the case of preterm labor and in the case of term labor, demonstrating that mast cell degranulation, and therefore their activation, activated uterine contractility [93]. PEA supplementation could be a valid preventive strategy to block premature activation of mast cells. In 1993, professor and Nobel prize winner in medicine, Rita Levi Montalcini, unveiled the mechanism of action of PEA, demonstrating that the molecule was able to restore the homeostasis of inflammatory processes because of its specific modulation and inhibition of mast cell degranulation [94]. Several scientific studies have demonstrated how PEA significantly reduces both the number of mast cells and the release of histamine, TNF-α, and prostaglandins [95,96,97]. Until now, no clinical research has examined the impact of PEA during pregnancy. Nonetheless, there is encouraging scientific data in this area, and we expect that more research will be performed soon. PEA is an endogenous component of amniotic fluid and fetal membranes; it may have an impact on all the inflammatory markers linked to premature labor, and moreover, it has no negative effects on the mother or the fetuses, as demonstrated by a recent in vivo study [98]. A micronized PEA formulation, the genetic and mammalian toxicology of which was evaluated through good laboratory practice (GLP) compliant toxicological investigations, which included the Ames test, in vitro mammalian cell micronucleus test, the acute oral up-and-down procedure, a 14-day toxicity study, and a 90-day toxicity study. The results revealed no evidence of mutagenicity, cytotoxicity, or genotoxicity. Additionally, results of the acute oral up-and-down procedure found the acute oral LD50 in rats to be >2000 mg/kg body weight. Furthermore, the 14-day (acute) and 90-day (subchronic) oral toxicity studies conducted on rats revealed no treatment-related adverse events and determined the no-observed adverse-effect level (NOAEL) of PEA, on rats, even at a high dose of 1000 mg/kg body weight/d (NOAEL), which translated to an HED, it corresponds to a dose of 9.7 g/d [98].

This prenatal developmental toxicity study contributes to opening new clinical applications for PEA. It indicates that PEA is well tolerated by and could be safe during pregnancy to prevent premature rats [98] (Table 4).

Despite the compelling biological profile of PEA, which exhibits anti-inflammatory, analgesic, neuroprotective, and mast cell-stabilizing effects through multiple molecular pathways, such as PPAR-α activation and the entourage effect with endocannabinoids, its clinical translation could be impeded by pharmacokinetic challenges [99]. PEA may have poor oral bioavailability, mostly caused by its high lipophilicity and low aqueous solubility. These properties could be an obstacle to converting the drug’s encouraging preclinical benefits into reliable clinical results. Otherwise, it could be easily bypassed through innovative formulations (such as micronizations, ultramicronizations) that could overcome solubility challenges. In any case, a dose of 200 mg should not have an absorption issue because it has the same absorption profile as micronized versions, as recently shown. Moreover, a strategy of double daily dosing could help maintain more stable therapeutic levels over time because of peak plasma concentrations [99].

### 3.5. Other Natural Strategies

#### 3.5.1. Alpha Lipoic Acid

Thioctic acid, also known as alpha-lipoic acid, or ALA, has gained more space as an effective clinical approach in cases of high-risk pregnancies and beyond [100].

ALA has both an immunomodulatory action (by suppressing the number and percentage of Th1 and Th17 cells, impairing the NK cell cytotoxicity, and increasing splenic T-regulatory (T-reg) cell count), and a potent anti-inflammatory action [101]. It inhibits, for instance, the NF-κB pathway and reduces IL-1β and IL-6 levels in different models, such as in human umbilical vein endothelial cells (HUVECs) and in vascular smooth muscle cells (VSMCs). [102,103]. Moreover, it counteracts other pro-inflammatory factors, such as TNF-α, IL-8, IL-17, and INF-ɤ [101].

Clinical evidence demonstrated, for instance, that in patients at risk of spontaneous abortion with subchorionic hematoma, oral ALA supplementation (600 mg), in association with vaginal P4 (400 mg), accelerated hematoma resorption with respect to the P4 alone [104]. Nineteen pregnant women between 20 and 40 years old, between 6th and 13th week of physiological pregnancy, with threatened miscarriage and ultrasound evidence of subchorionic hematoma, with pelvic pain and/or vaginal bleeding, were included in a randomized controlled trial. Enrolled women were divided into two groups: the control group was treated with 400 mg P4 (200 mg 2 times per day), given by vaginal suppositories, and the case study group was treated with the same P4 dosage, plus ALA, given orally at the dose of 600 mg [104]. Although the improvement was general, a very different time trend in the healing was evident and was clearly much faster in patients treated with ALA plus P4 (*p* < 0.05). Moreover, uterine contractions and other faster symptoms disappeared in ALA treated group [104].

The same effect was also achieved in the case of vaginal ALA [105]. Pregnant women at age of 24–40 years and in the 7th to 12th week of physiological gestation, with pelvic pain and with or without moderate vaginal bleeding, and subchorionic hematoma, were randomized to receive 400 mg vaginal P4 or 10 mg ALA plus P4, until resolution of the clinical picture. Also in this case, the ALA plus P4 group had a faster hematoma resorption (*p* < 0.05) and full remission of pelvic pain and vaginal bleeding, in the first 20 days of treatment [105].

According to the National Institute of Child Health and Human Development Maternal–Fetal Medicine Unit Network, cervical length (CL) below 25 mm represents a risk factor for PTB, both in low- and high-risk pregnancies [106]. ALA supplementation may also prevent CL shortening associated with uterine contractions [107].

In an open-label randomized controlled pilot study, a total of 122 women attending the first-trimester aneuploidy screening at 11–14 weeks of pregnancy, aged between 18 and 41 years, and presenting risk factors for PTB, were enrolled and treated with 600 mg oral ALA. Data were compared with a control group, followed by standard practice until delivery without any additional supplementation. ALA supplementation stabilized CL changes during the first trimester of pregnancy compared to the control group, which otherwise had a significant decrease in CL (*p* < 0.05). As a result, hospital admissions for threatened PTB significantly decreased compared with the control group (3.4% vs. 14.3%; *p* = 0.03) [108]. A retrospective study analyzed data from 300 pregnant women, at 14–34 weeks of gestation, with premature uterine contractions, but no vaginal infections, and a history of miscarriage and premature delivery [108]. The incidence of preterm uterine contractions and hospitalization rates decreased when magnesium and ALA were administered beginning in the fourteenth week of pregnancy, according to the results [108]. ALA supplementation may also prevent preterm premature rupture of the fetal membranes (pPROM) as evidenced by an in vitro model, in which ALA inhibited TNF-α and thrombin-induced fetal membrane (FM) weakening in a dose-dependent manner [109]. Despite the efficacy of ALA in maintaining uterine quiescence and counteracting premature uterine contractility in high-risk pregnancies, it is important to outline that several data reports that ALA has a short half-life and bioavailability of about 30%, due to its hepatic degradation, reduced solubility and instability in the stomach [110].

#### 3.5.2. Vitamin B9 Folic Acid

Folic acid is well known in pregnancy for the prevention of neural tube defects. Folic acid supplementation during pregnancy has been linked to a lower risk of unfavorable outcomes, including preterm birth, according to systematic reviews and meta-analyses. However, a significant effect on pre-term delivery was only seen when folic acid was initiated during the pregnancy and not with pre-conception supplementation, though the trend was still apparent [111].

#### 3.5.3. Omega-3

In addition to being vital for many facets of health, omega-3 fatty acids are crucial for the structural integrity of cell membranes, and many studies have linked optimal omega-3 intake with positive fetal and maternal outcomes. Supplementation has also been demonstrated to lengthen gestation and lower the risk of early or preterm delivery in a number of reviews and meta-analyses [112,113].

## 4. Discussion

**Figure 1:**
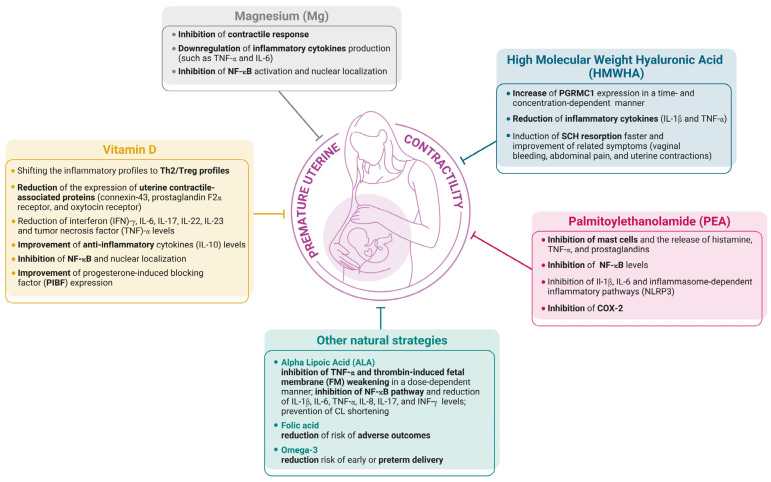
Synergic activity of natural molecules to counteract premature uterine contractility.

The maintenance of uterine quiescence throughout gestation is a highly orchestrated process involving a delicate balance between immunological tolerance, hormonal regulation, and mechanical adaptation. As highlighted in this review, premature activation of the myometrium—often driven by inflammatory cascades—remains a leading cause of preterm birth, with substantial perinatal morbidity and mortality. While standard therapeutic strategies, such as tocolytics and progesterone supplementation, have shown inconsistent efficacy and notable limitations, increasing attention is being devoted to natural molecules with pleiotropic and synergistic actions targeting multiple pathways simultaneously (Figure 1).

Vitamin D, HMWHA, and magnesium each contribute to uterine quiescence through distinct yet complementary mechanisms. Vitamin D emerges not only as an immunomodulator—promoting maternal tolerance and enhancing PIBF expression—but also as a direct inhibitor of contractile-associated proteins (e.g., connexin-43, oxytocin receptor) and pro-labor mediators such as CRH and COX-2. Critically, its action on the NF-κB pathway appears central to prevent the inflammatory shift that initiates labor [41,43]. Clinical evidence consistently supports its supplementation as protective against PTB, with recent meta-analyses indicating up to a 30% risk reduction [49].

HMWHA, beyond its well-established role as a structural component of the extracellular matrix, has demonstrated functional relevance in sustaining progesterone signaling—specifically through upregulation of PGRMC1, a non-classical progesterone receptor whose downregulation is associated with preterm labor and PPROM [56,57]. Its ability to stabilize cervical architecture, inhibit TLR-mediated inflammation, and synergize with other molecules (e.g., Mg, ALA, vitamin D) in clinical cohorts [50,51] supports its integration into multi-target preventive regimens, particularly in early pregnancy complications like subchorionic hematoma.

Magnesium, with its dual capacity to inhibit myometrial calcium signaling and suppress NF-κB-driven cytokine production (e.g., TNF-α, IL-6), acts as a fundamental anti-inflammatory and spasmolytic agent in preventive strategies [70,71]. Epidemiological and interventional data further link adequate Mg status to reduced PTB risk, reinforcing the need for routine assessment and supplementation—especially given widespread dietary insufficiency.

Perhaps the most innovative insight of this review concerns PEA, an endogenous N-acylethanolamine whose role in obstetrics remains underexplored despite compelling mechanistic rationale. PEA acts on multiple levels: inhibiting NF-κB activation, downregulating NLRP3 inflammasome activity, suppressing COX-2 and prostaglandin synthesis, and—most notably—stabilizing mast cells [82,83,96]. Given that mast cell degranulation directly enhances uterine contractility via histamine and prostaglandin release, PEA’s ability to modulate this process represents a novel, non-hormonal avenue to preserve quiescence. The observation that endogenous PEA levels are elevated in women who deliver preterm may reflect a compensatory anti-inflammatory response [81], supporting the hypothesis that exogenous PEA administration could “top-up” this system before decompensation occurs. Importantly, preclinical safety data—including a high NOAEL and absence of teratogenicity [98] strongly suggest PEA’s suitability for use in pregnancy.

The convergence of these natural molecules on common pathways—particularly NF-κB, IL-1β/IL-6 signaling, prostaglandin synthesis, and mast cell stabilization—suggests a synergistic potential. Rather than targeting a single node in a complex network (as do most tocolytics), a multi-component nutraceutical strategy could restore homeostatic resilience more effectively, particularly in high-risk or inflammation-driven phenotypes of PTB. This is corroborated by already available clinical studies where combinations of HMWHA, Mg, ALA, vitamin B6, and vitamin D significantly exerted a synergic activity, by reducing uterine contractions, hospitalization, and PTB rates compared to standard care.

Nevertheless, several limitations must be acknowledged, and robust randomized controlled trials (RCTs) specifically evaluating combinations of these molecules—versus monotherapies or placebo—need to be performed in the future.

In conclusion, integrating vitamin D, HMWHA, magnesium, and PEA into a unified strategy may offer a promising, low-risk approach to maintaining uterine quiescence. Future research should prioritize well-designed RCTs testing such multi-target formulations, ideally with mechanistic endpoints (e.g., cytokine profiling, NF-κB activity in peripheral cells, cervical remodeling markers), to translate this conceptual framework into evidence-based clinical practice.

## Figures and Tables

**Table 1:** The role of vitamin D in preventing premature uterine contractility. Experimental and clinical evidence.

Study	Model and Design	Interventions	Findings	Ref.
Ribeiro, V.R. 2022	Peripheral blood mononuclear cell (PBMC) from 40 pregnant women	100 nM of vitamin D for 24 h	Shifting the inflammatory profiles, to Th2/Treg profiles; decreasing levels of interferon (IFN)-γ, IL-6, IL-17, IL-22, IL-23 and tumor necrosis factor (TNF)-α; increasing levels of anti-inflammatory cytokines IL-10 and TGF-β	[38]
Szekeres-Barthó, J. 2020	Phytohemagglutinin (PHA)-activated peripheral lymphocytes	Escalating levels of vitamin D and a standard dose of progesterone for 24 h	Increases progesterone-induced blocking factor (PIBF) expression	[37]
Thota, C. 2014	Uterine myometrial smooth muscle (UtSM) cells	0, 5, 10, 50, 150, and 300 nmol/L of 1,25 (OH)_2_ vitamin D for 24 h	Inhibition of the expression of uterine contractile-associated proteins (connexin-43, prostaglandin F2α receptor, and oxytocin receptor); inhibition phosphorylation of IkBα and nuclear translocation of NFkB-p65; decrease in the expression of IL-1β, IL-6, and -13 and TNFα	[41]
Wang, B. 2018	Placenta from healthy women with estimated gestational age of 38 and 40 weeks	1 nM, 10 nM, 10 µM of vitamin D for 24 h	Inhibition of CRH (Corticotropin-releasing Hormone) and COX-2 genes by upregulating miR-181 b-5p or miR-26b-5p	[43]
Singh, J. 2017; Zhou, S.S. 2017; Moghib, K. 2014	Pregnant women	Vitamin D supplementation until the end of pregnancy	Reduction in PTB	[45,47,49]

**Table 2:** The role of high molecular weight hyaluronic acid (HMWHA) in preventing premature uterine contractility. Experimental and clinical evidence.

Study	Model and Design	Interventions	Findings	Ref.
Zhao, G. et al., 2014	Granulosa cells (in vitro study)	HMWHA (100 μg/mL, 200 μg/mL, and 500 μg/mL)	HMWHA increases PGRMC1 expression in a time- and concentration-dependent manner	[53]
Cilaker Micili, S. et al., 2023	Rats (in vivo study)	Low dose (2.5 mg) and high dose (5 mg)	HMWHA prevents PTB and decreases inflammatory cytokines (IL-1β and TNF-α)	[63]
Parente, E. et al., 2023	Pregnant women (clinical study)	HMWHA (200 mg) in association with natural molecules versus control group	HMWHA prevents PTB and other adverse events (pelvic pain, spontaneous contractions, miscarriages, and hospitalization)	[50]
Porcaro, G. et al., 2024	Pregnant women (clinical study)	HMWHA (200 mg) in association with natural molecules in association with vaginal P4 versus control group	HMWHA induces SCH resorption faster and improves related symptoms (vaginal bleeding, abdominal pain, and uterine contractions)	[51]

Abbreviations: POI = primary ovarian insufficiency; PTB = preterm birth; SCH = subchorionic hematoma; and P4 = progesterone.

**Table 3:** “The role of magnesium (Mg) in preventing premature uterine contractility. Experimental evidence”.

Study	Model and Design	Interventions	Findings	Ref.
Fomin, V.P. 2005	Pregnant human myometrial strips	Magnesium Sulfate (MgSO_4_) 5 mmol/L for 20 min	Inhibition contractile response	[70]
Sugimoto, J. 2012	In vivo and in vitro mononuclear cells	2.5 mM MgSO_4_ 1, 2, 4 h following LPS stimulation	Downregulation of inflammatory cytokines production, such as TNF-α and IL-6; reduction in NF-κB activation and nuclear localization	[71]

**Table 4:** Relevant activities of palmitoylethanolamide (PEA).

Study	Model and Design	Findings	Ref.
D’Agostino, G. 2009; Di Paola, R. 2016	In vivo model	Inhibition of NF-kB levels	[82,83]
Motomura, K. 2022	In vivo model	Inhibition of TNF-α, Il-1β, IL-6, inflammasome-dependent inflammatory pathways (NLRP3)	[84]
Esposito, G. 2014	In vitro model	Inhibition of COX-2 and prostaglandins	[85]
Cerrato, S. 2010	In vitro model	Inhibition of mast cells and the release of histamine, TNF-α, and prostaglandins	[96]

Abbreviations. NF-kB = Nuclear factor-κB; TNF-α = Tumor necrosis factor alpha; Il-1β = Interleukin-1β; IL-6 = Interleukin-6.

## Data Availability

No new data were created.

## Abbreviations

The following abbreviations are used in this manuscript:

AEA	anandamide
ALA	alpha lipoic acid
CL	cervical length
COX-2	cyclooxygensae-2
CRH	corticotropin-releasing hormone
CX-43	connexin 43
FOXP3	forkhead box 3
GLP	good laboratory practice
HCA	histological chorioamnionitis
HUVECs	human umbilical vein endothelial cells
IFN-γ	interferon gamma
IL-1β	interleukin 1β
IL-6	interleukin 6
LPS	lipopolysaccharide
MC	mast cells
MgSO4	magnesium sulfate
MMP-9	matrix metalloproteinase 9
NAE	N-acyl-ethanolamine
NF-κB	nuclear factor-kappa B
NK	natural killer
NLRP3	NOD-, LRR- and pyrin domain-containing protein 3
NO	nitric oxide
PGF2	prostaglandins
PGRMC1	progesterone receptor membrane component 1
PHA	phytohemagglutinin
PIBF	progesterone-induced blocking factor
POI	primary ovarian insufficiency
PPAR-α	proliferator-activated receptor
PPROM	preterm premature rupture of membranes
PTB	preterm birth
RCTs	randomized controlled trials
SCH	subchorionic hematoma resorption
TLRs	toll-like receptors
TNF-α	tumor necrosis factor
Treg	T-regulatory
TRPV1	vanilloid receptor 1
VSMCs	vascular smooth muscle cells
